# The SÖderberg Socket 2.0: A Technical Note

**DOI:** 10.33137/cpoj.v2i2.33505

**Published:** 2020-03-10

**Authors:** B Söderberg, G Guerra, T Fagerstrom, K Permpool, S Phaipool

**Affiliations:** 1 Centre of Excellence for Prosthetics and Orthotics, Scandinavian Orthopaedic Laboratory, Bangkok, Thailand.; 2 Sirindhorn School of Prosthetics and Orthotics, Faculty of Medicine, Siriraj Hospital, Mahidol University, Bangkok, Thailand.

**Keywords:** Prosthesis, Transtibial Amputees, Amputation, Transtibial socket, Prosthetic suspension

## Abstract

**BACKGROUND:**

Transtibial prosthesis socket trim lines have remained fairly consistent over the past decade, and based on methods such as a supracondylar cuff suspension. However, with vacuum suspension methods, trim lines can change.

**OBJECTIVE:**

An objective of this technical note was to inform practitioners how to fabricate a socket in a better way. A step-by-step fabrication guide is provided for the prosthetist.

**METHODS:**

A unilateral transtibial amputee was selected for this technical note. We provide a detailed description of the different steps of fabrication as well as patient feedback. The fabrication involved fabrication of a vacuum socket using Pre-preg carbon fiber and anti-bacterial Ethylene-Vinyl-Acetate (EVA), as a proximal flexible brim.

**FINDINGS:**

The properties of EVA and Pre-preg carbon fiber allow for fabrication of a transtibial socket with a flexible proximal brim. The new design resulted in greater comfort and increased range of motion in the patient studied. The patient subjectively noted enhanced squatting and cycling capabilities while using the updated socket and flexible proximal brim.

**CONCLUSION:**

This technical note presented a fabrication guide for a new style of socket and preliminary patient feedback. Clinical studies evaluating functional and biomechanical effects of this new socket design are needed.

## INTRODUCTION

Prosthetic interface, socket technologies and suspension variants have evolved in recent years to allow for a greater adjustability of the prosthesis to the extremity. Numerous transtibial prosthesis technologies work to facilitate an improved dynamic fitting or aid the overall function of the individual.^1^ Various modifications to the socket can augment suspension characteristics and even assist gait of the user.^2^ Some companies have focused on utilizing ratcheting technologies to aid in adjustability of the prosthesis socket, such as the BOA system (Click Medical, BOA, Steamboat Springs, Colorado, USA). In this same vein, it is equally important to augment the range of motion for individuals with specific seating, cycling, kneeling and walking requirements. Trim lines have remained consistent in the literature and exploration of various socket trim lines might offer patient functional improvements.

Transtibial prosthesis wearers participating in cycling or squatting and seating might benefit from an enhanced knee range of motion. This is also true when sitting down, for example, in a chair or a car seat. Traditional transtibial socket trim lines extend the medial and lateral walls proximally over the epicondyles.^3^ The rationale for doing so is rooted in habits and seldom questioned. Higher trim lines of the supra-condylar and Patella Tendon Bearing (PTB) styles were previously justified because of the introduction of the supracondylar suspension and to enhance medial lateral stability. Anterior-posterior and medial-lateral trim lines are rigid and encompass the proximal aspect of the socket in the below knee prosthesis. This conventional design has still remained, even if supracondylar and cuff suspension methods have shifted towards vacuum.^1^ Recent transtibial socket clinical guidelines have served to aid the prosthetist decision making process by offering greatly needed recommendations.^4^ Such as using vacuum suspension as a viable alternative to supracondylar and cuff suspensions.

Our previous Söderberg socket 1.0 research evidenced that a lowered trim line design can mitigate excessive motion at the knee whilst still allowing the epicondyles to travel anteriorly out of the proximal walls during knee flexion.^5^ In our clinic we have witnessed the success of this socket design for users with recreational lifestyles performing activities such as cycling and hiking. More recently, the initial Söderberg socket 1.0 design presented in 2001 has been adjusted, and much of the rigid socket is now replaced with a flexible integrated proximal socket brim and trim line. It has been our clinical experience, that this additional removal of material when combined with elevated vacuum and a reduced trim line, elicits a greater range of knee motion, protects the suspension sleeve from breaking and can afford a shorter residual limb these same benefits.

It was the purpose of this technical note to reintroduce an updated version of the Söderberg socket design and provide preliminary patient feedback. We questioned whether these updates would be viewed as a useful benefit to a patient with a desire for more range of motion.

## METHODOLOGY

The patient for the Söderberg 2.0 socket is typically a transtibial amputee with a desire for enhanced range of motion during sitting, squatting or cycling or for aesthetic reasons during sitting. Patients with a short transtibial residual limb can also potentially benefit from the elevated flexible brim for better suspension. Often times the user is already wearing an endoskeletal modular prosthesis with liner and sleeve aided passive suspension.

One active male participant was recruited (65 years, 180cm, 87kg) K3 functional level,^6^ with residual limb length of 29cm mid-patella tendon to distal end of residual limb. The cause of amputation was trauma at age 55 and prior prosthesis socket and experience was with a pin lock suspension, total surface bearing style prosthesis, endoskeletal design and dynamic response foot. This patient had previously expressed a dislike of traditional socket trim lines, especially during daily trail cycling. Without objection, he agreed to volunteer for custom fitting of the Söderberg 2.0 socket. This single case record was granted approval through policies of the ethical committee at the Faculty of Medicine, Siriraj Hospital, Mahidol University. Our sampling method for this particular study was a convenience sample, and the participant was recruited from our clinic by word of mouth. As the participant was a healthy ambulator with a high functional level and a unique need, he was recruited for the study. Moreover, the patient had no other underlying health conditions or activity restrictions which would exclude them from participation.

### Casting and rectification

**Figure 1:**
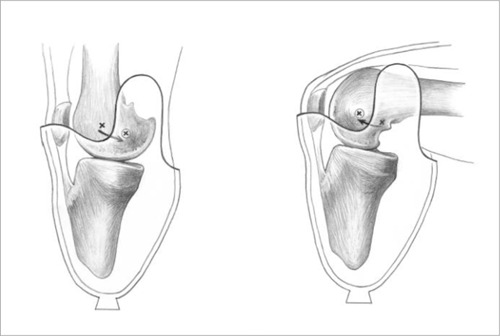
Illustration demonstrating femoral movement within the Söderberg socket.^5^

**Figure 2:**
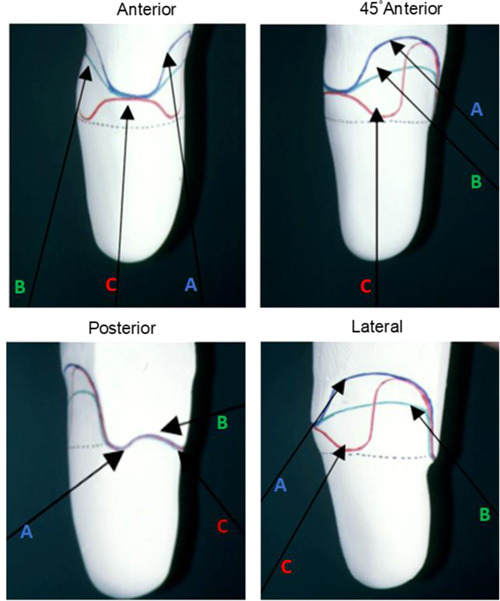
Images illustrating the anterior, 45° anterior, posterior view as well as lateral views of the SOL style positive plaster model. Where (**A**) indicates trim lines for supracondylar suspension, (**B**) trim line for a PTB type socket with cuff suspension, and (**C**) trim line for a Söderberg type socket.

The patient first donned a Simplicity tapered PUR liner (Otto Bock, Germany), the residuum was covered with protective wrap and bony landmarks were drawn with an indelible marker. The medial and lateral femoral epicondyles were marked, and a trim line across the proximal limb was drawn. An outline of the tibia and fibular head was defined, and any anatomical points of interest were noted for later rectification. The prosthetist performed a routine evaluation of the patient and created a Plaster of Paris positive cast of the residual limb using a total surface bearing (TSB) technique. We recommend to extend the proximal trim lines of the cast well over the condyles (3-5cm) as these higher than traditional trim lines provide landmarks which can later be identified during rectification. A two-stage cast was created, firstly, an anterior slab was made to capture the head of the fibula, patella and tibial crest in 30° of knee flexion. This initial flexion permits easier definition of the anatomy of the tibia and patella tendon. Secondly, a circumferential plaster wrap was provided to encapsulate limb volume, posterior wall, and the entirety of the limb was cast in 10-20° knee flexion. These varying casting angles are based off of experience and are modifications of previously established stage casting methods. Flexion in the first stage allows for capturing the bony aspects of the limb.^7^ During the second stage of casting it also important to capture the remaining residual muscle activity by asking the patient to contract and relax numerous times. Cast rectification was then performed using traditional principles of the TSB prosthesis with PUR liner.^8^ A sketch, as well as illustration of the Söderberg socket trim lines is provided in order to give a better understanding of the socket concept in Figure 1 and Figure 2.

### Fabrication and fitting

Diagnostic (clear check socket) prosthesis fabrication was manufactured using a 12mm thick Northplex square sheet (North Sea Plastics Ltd, Glasgow, Scotland). The socket was aligned using modular componentry, a one-way expulsion valve was added to the socket and patient was fit with VAS, knee sleeve, and a PUR liner (Otto Bock, Germany) to create vacuum suspension. This suspension was selected based on the need for an improved connection between the residual limb and socket, to reduce volume fluctuations,^9^ and because of the socket’s less rigid trim lines.^10^ Typical below knee socket designs are higher along the lateral and medial aspects in order to provide medial lateral support around the condyles. The anterior trim line runs along the mid-patella region or slightly below as to not restrict movement. Posteriorly, the trim line allows both hamstring tendons to move freely and travels slightly proximal to the posterior compartment. The previous Söderberg design trim lines did not have the added advantage of a flexible proximal brim. At this stage, the patient donned the check socket and trim lines were evaluated and trimmed to provide maximum medio-lateral and rotational stability as well as comfort according to the Söderberg 1.0 trim line.^5^ Each patient will present with individual needs, however, we recommend reducing trim lines on the rigid socket approximately 1-2cm. The patient ambulated and was evaluated to confirm socket fit, alignment and patient comfort. The check socket will allow for minor adjustments, though this was not required in this patient. Finally, a new circular cast was performed with the patient wearing the check socket to capture the proximal portion of the knee. The check socket, now with extended plaster cast, was filled with plaster and rectified into an updated model.

The definitive fabrication was split into two processes; the first being the fabrication of the proximal brim and the second being creation of the distal definitive socket. To do so, an antibacterial ethylene-vinyl-acetate (EVA) material (Agruquero, Madrid, Spain) was formed over the proximal part of the updated positive model. This material self-adheres well and leaves no seams after joining. The oven setting temperature for this material was 140º Celsius for 10 minutes and model set time was 20 minutes. After the brim was set, it was removed and trimmed to a proper thickness and skived down along the distal areas to allow for a good integration between the layers of carbon fiber. Upon completion of the proximal brim, a second process occurred to create the distal portion of the definitive socket. The distal portion was made from multiple layers of preimpregnated with resin (Prepreg) carbon fiber. The pregreg carbon fiber must be stored at a temperature of -20°C prior to curing. Storing the material at this temperature extends the life of the material to about one year.^11^

**Figure 3:**
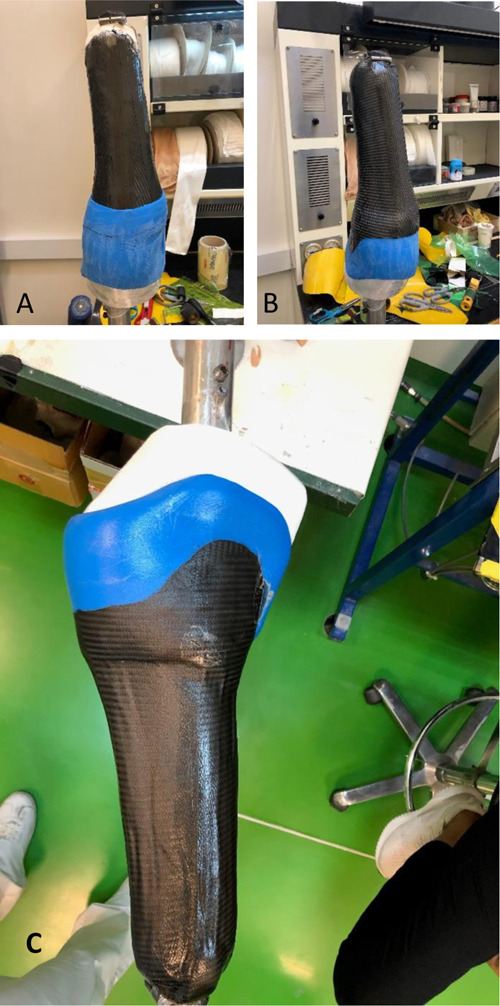
Images illustrating the (a) proximal brim over the first layer of prepreg carbon fiber, (b) trimmed version with second layer of prepreg carbon fiber applied and (c) final socket after curing.

Preparation of the positive model for the Prepreg requires removal of the proximal brim. A PVA bag is then drawn over the model in the same method as in traditional resin lamination. Prepreg strips were then individually adhered onto the model, layer by layer, whilst controlling fiber directions to achieve the desired design and strength. A total of 4 layers of carbon fiber were then wrapped circumferentially in an evenly distributed manner. The proximal brim was then placed back onto the model and finally an additional 4 layers of Prepreg were wrapped along the brim transition point. This sandwiching allows flexibility of the proximal portion of the socket. The transition from the EVA to the carbon fiber will show no ridges as this EVA material adheres to the prepreg carbon fiber in the definitive socket uniformly. Moreover, a oneway expulsion valve was placed between the model, Prepreg and distal socket adapter. A second PVA bag was then applied to enable vacuum suction during curing. The socket was then placed in an oven overnight for curing per Prepreg manufacturer recommendations (Figure 3).

Initial cutting and trimming of the socket were performed after the device was assembled. The patient donned the device and flexible proximal trim lines as well as alignment were assessed and adjustments made. Trim lines were trimmed to permit full range of motion during the clinic visit. The patient returned to daily activities, and over the course of a year, was asked to return to his typical cycling, hiking and rock-climbing activities and to informally record subjective feedback with respects to range of motion, stability and comfort. The patient returned to living abroad, and subjective feedback from the patient was evaluated in person at the clinic a year later.

## RESULTS

The new Söderberg 2.0 socket technical achievement is in its ability to blend two different materials together in a socket which enhanced user function. Subjective feedback was received from the patient in person at the clinic. Although terse, feedback was noteworthy and useful, with the patient describing his cycling and daily activities as “enhanced and without restriction in the knee”. The user expressed that they were able to cycle as often as they wished and do so without any discomfort or restriction of motion. On a side note, he also reported that the silicone knee sleeve had lasted longer than with his previous sockets.

## DISCUSSION

This technical note explored a new socket design using a combination of materials that improved both range of motion and comfort for the transtibial prosthesis user. Our technical achievements were seen in the ability to seamlessly integrate two new materials together in a single rigid-flexible socket design. This technique proved beneficial for the patient during activities of daily living.

**Figure 4:**
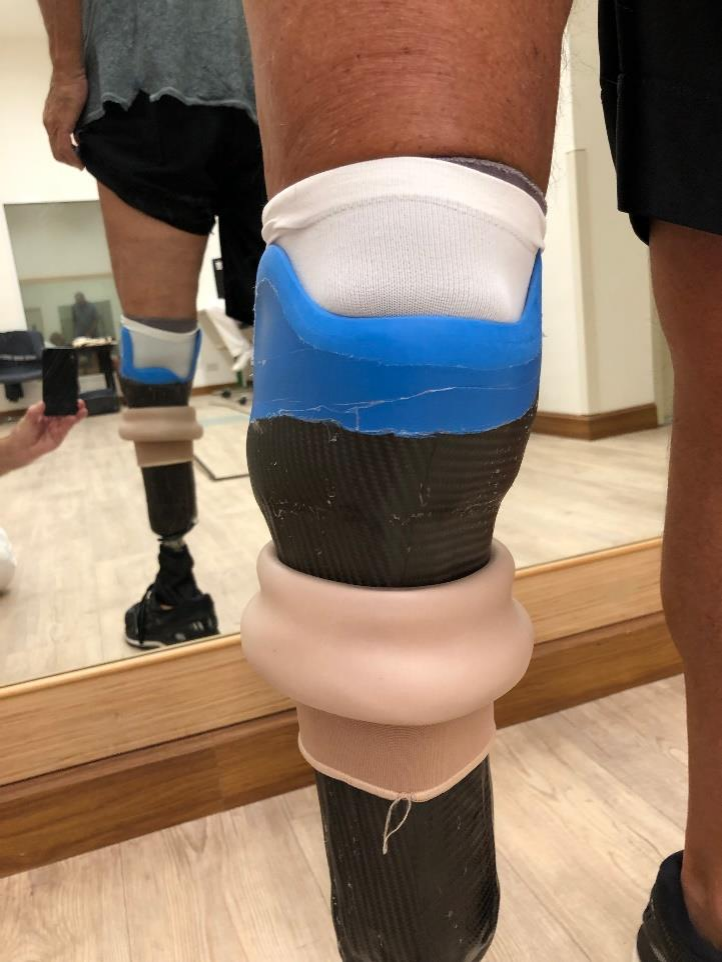
Image of the patient wearing the Söderberg 2.0 prosthesis.

The previous Söderberg socket design does not provide the user with the superior proximal socket comfort and sleeve protection offered in the current socket style. The patient noting increased lifespan of the liner was most likely due to the flexible brim reducing strain on the sleeve, whereas a regular hard socket brim might not have been able to do so. Although, outside of the purview of this technical note, a more robust set of research outcome measurements and comparison between traditional sockets could provide further insight. Due to a small sample size, care should be taken not to generalize current findings. In addition, this user’s residual limb length was longer than average which might have affected his feedback of the device. In Figure 4, we provide an image of the patient fit with the Söderberg 2.0 prosthesis.

A potential caveat of this socket design is the requirement of costly Prepreg technology, however, this material is critical because of the rigid socket’s seamless interweave of the EVA flexible brim. Prepreg carbon fiber is widely used in P&O clinics and we believe that this technical note demonstrates a new option in manufacturing of prosthetic sockets that can further improve quality of life for the user. The positive subjective results provided by the patient, encourages us to further evaluate the technique and hopefully, with future clinical studies, the Söderberg 2.0 socket design will add to available options for the transtibial prosthesis user.

## DECLARATION OF CONFLICTING INTERESTS

The authors have no conflicts of interest to declare.

## ETHICAL APPROVAL

This single case record was granted approval through policies of the ethical committee at the Faculty of Medicine, Siriraj Hospital, Mahidol University.

## AUTHOR CONTRIBUTION

- **Bengt Söderberg**,
  conceived the idea for the project, supported technical aspects and contributed to the writing of the manuscript
- **Teddy Fagerstrom**,
  conceived the idea for the project, supported technical aspects and assisted in the writing of the manuscript
- **Gary Guerra**,
  supported the technical aspects and contributed to the writing of the manuscript
- **Kwannate Permpool**,
  supported the technical aspects and contributed to the writing of the manuscript
- **Sarawanee Phaipool**,
  supported the technical aspects and contributed to the writing of the manuscript

## SOURCES OF SUPPORT

The authors received no financial support or assistance for this research.
